# Lipocalin 2: A New Antimicrobial in Mast Cells

**DOI:** 10.3390/ijms20102380

**Published:** 2019-05-14

**Authors:** Yu-Ling Chang, Zhenping Wang, Satomi Igawa, Jae Eun Choi, Tyler Werbel, Anna Di Nardo

**Affiliations:** Department of Dermatology, University of California, San Diego, La Jolla, CA 92093, USA; ychangucsd12@gmail.com (Y.-L.C.); zhenping.w@gmail.com (Z.W.); dcebr0615@gmail.com (S.I.); dermagrace79@gmail.com (J.E.C.); TWerbel@ucsd.edu (T.W.)

**Keywords:** mast cell, lipocalin 2, antimicrobial, microbiome, sphingosine-1-phosphate cathelicidin, keratinocyte

## Abstract

Mast cells (MCs) play a significant role in the innate immune defense against bacterial infection through the release of cytokines and antimicrobial peptides. However, their antimicrobial function is still only partially described. We therefore hypothesized that MCs express additional antimicrobial peptides. In this study, we used FANTOM 5 transcriptome data to identify for the first time that MCs express lipocalin 2 (LCN2), a known inhibitor of bacterial growth. Using MCs derived from mice which were deficient in LCN2, we showed that this antimicrobial peptide is an important component of the MCs’ antimicrobial activity against *Escherichia coli* (*E. coli*). Since sphingosine-1-phosphate receptors (S1PRs) on MCs are known to regulate their function during infections, we hypothesized that S1P could activate LCN2 production in MCs. Using an in vitro assay, we demonstrated that S1P enhances MCs antimicrobial peptide production and increases the capacity of MCs to directly kill *S. aureus* and *E. coli* via an LCN2 release. In conclusion, we showed that LCN2 is expressed by MCs and plays a role in their capacity to inhibit bacterial growth.

## 1. Introduction

Mast cells (MCs) are known to be key mediators of allergic inflammation in peripheral tissues. However, given that MCs play a critical role in protection during acute infection, they are also considered to be primary inducers of both innate and adaptive immune responses [1,2]. MCs, when activated in response to pathogens, secrete preformed cytokines that facilitate the development, amplification, and regulation of the innate immune response. Current literature supports the concept that MCs express different innate immune receptors, such as Toll-like receptors (TLRs), that initiate pathogen recognition [3,4]. MCs can be activated to directly kill pathogens by phagocytosis or through antimicrobial peptide (AMP) release [5,6]. We have previously published that during bacterial or viral encounters MCs secrete cathelicidin, an AMP, and directly kill bacteria and viruses [7,8]. Cathelicidin AMP (CAMP) has been broadly studied in innate host defense. CAMP is the only human, cathelicidin-derived AMP that is present in MCs [9]. The expression of cathelicidin is classically induced by the Toll-like receptor 2 (TLR2) pathway, which is triggered by various ligands, including lipoteichoic acid (LTA) [10]. 

While CAMP in MCs has been extensively studied, other antimicrobial proteins have not been equally investigated. In fact, while it is known that the antimicrobial function of MCs in the skin is primarily derived from the activation of the cathelicidin family of AMPs [9], cathelicidin-deficient murine MCs have residual antimicrobial capacity [9]. Based on a transcriptome analysis of human MCs, FANTOM 5 data [11], we found that lipocalin 2 (LCN2), a molecule that binds to bacterial siderophores inhibiting bacterial growth [12], is present in MCs. 

In the present study, we show for the first time that LCN2 is secreted by MCs and enhances their antimicrobial function. 

## 2. Results

### 2.1. TLR4 Ligand LPS, a Gram-Negative Bacteria Byproduct, Induces LCN2 Expression and Production in both Human and Mouse MCs

**Figure 1:**
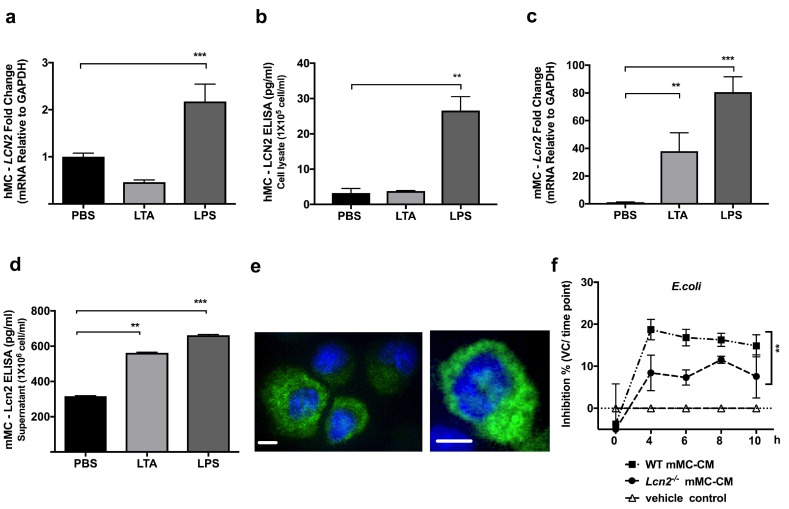
Bacteria ligands induced Lcn-2 expression and production in both human and mouse mast cells (h- and mMCs). hMCs or mMCs were stimulated with control Phosphate-buffered saline (PBS), 10 μg/mL lipoteichoic acid (LTA), or 100 ng/mL lipopolysaccharide (LPS) for 4 (for q-PCR) or 24 (for ELISA) hrs. (**a**) hMC LCN2 expression was measured by q-PCR, and (**b**) hMC LCN2 production was quantified by ELISA. (**c**) mMC *Lcn2* expression was measured by q-PCR, (**d**) mMC Lcn2 production was quantified by ELISA. (**e**) Anti-Lcn2 (green) and DAPI (blue) immunofluorescence staining of mMCs and its amplified image (the right panel) demonstrated that mMC Lcn2 is expressed in both granule and cytoplasm. The scale bar is 10 µm (**f**) WT or *Lcn2*^−/−^ derived mMC-CM were collected for antimicrobial liquid assays against *E. coli*. Bacteria growth was measured by OD600. Data was shown by *E. coli* inhibition % calculated by the formula shown in materials and methods. **: *p* < 0.01, ***: *p* < 0.001.

To confirm that MCs express another unreported antimicrobial molecule, we first utilized FANTOM 5 transcriptome data [11] and screened for possible candidates. Transcriptome analysis showed that dermal hMCs express LCN2, and interestingly, it is expressed at higher levels in the skin MCs compared to in culture-expanded MCs. Expression was measured in transcripts per kilobase million (TPM) (Figure S3). Next, we investigated the expression of LCN2 mRNA and protein levels in hMCs and mMCs (Figure 1a–d). As shown in Figure 1, we confirmed that LCN2 is expressed in both human and mouse MCs as mRNA as well as proteins. To verify that LCN2 responds to bacterial stimulation, both hMCs and mMCs were stimulated with PBS, LTA (TLR2 ligand and gram-positive byproduct), or LPS (TLR4 ligand and gram-negative byproduct), and were measured at different time points. Data showed that LPS induces an increase of LCN2 mRNA expression in hMCs (Figure 1a) as well as an increase in its protein level (Figure 1b), while stimulation with LTA did not. Next, we validated Lcn2 expression and production in mMCs (Figure 1c, d). As data demonstrated, mMCs express Lcn2 and, when stimulated with LTA and LPS, highly increase *Lcn2* mRNA (Figure 1c) as well as Lcn2 protein production (Figure 1d). In addition, we evaluated Lcn2 by using immunofluorescence staining to confirm the protein expression and investigate Lcn2 localization in mMCs. As the immunofluorescence images demonstrated, mMCs expressed some Lcn2 at baseline level, and the magnified image demonstrated that Lcn2 is located in mMC cytoplasm (Figure 1e).

### 2.2. Lcn2^-/-^ MCs Kill E. coli Less Efficiently

Since it is already known that *Lcn2*^−/−^ mice are more sensitive to gram-negative bacteria [12,13], we sought to investigate the role of Lcn2 in the mMC response to gram-negative bacteria. To evaluate this response, we cultured mMCs derived from WT, and *Lcn2*^−/−^ mice and collected the culture medium for antimicrobial liquid assays. To compare baseline responses, the two types of mMC-CM from WT and the *Lcn2*^−/−^ mouse were incubated with PBS alone for 48hrs. The liquid assay demonstrated that the antimicrobial capacity of *Lcn2*^−/−^ mMCs against *E. coli* is significantly decreased compared to WT mMCs (Figure 1f). These data suggest that Lcn2 in mMCs plays a role in protection against *E. coli*.

### 2.3. S1P Increases the Release of LCN2 and CAMP from Mast Cells

While “in vitro” differentiated mMCs display AMP activity in response to the activation of TLR2 or TLR4 by bacteria byproducts such as LTA or LPS [14], “in vivo” dermal mMCs internalize TLR2 [15,16]. Without TLR2 expression, in vivo dermal mMCs still have AMP producing activity [15]. This report indicates that mMCs are using other receptors to mediate the response to gram-positive bacteria. Among those receptors, Sphingosine 1-phosphate receptors (S1PRs) play a critical role in regulating MC function during infections [8] by triggering MCs release of AMPs. S1P, the ligand of S1PRs, is a bioactive lipid mediator produced by multiple skin cells, such as keratinocytes, endothelial cells, and virus-activated MCs [8,17,18,19]. Based on these observations, we investigated the LCN2 response to S1P in MCs and compared it to the cathelicidin response (Camp was known to respond to S1P in MCs [8]).

**Figure 2:**
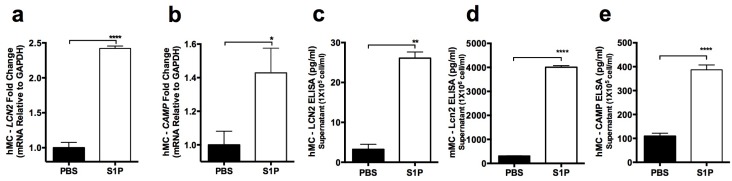
Sphingosine 1-phosphate (S1P) activates MCs and induces antimicrobial peptide (AMP) production of both LCN2 and Cathelicidin AMP (CAMP). hMCs and mMCs were stimulated with PBS (control) or 200 nM S1P for 4 (for q-PCR) or 24 (for ELISA) hr. (**a**) Human LCN2 and (**b**) CAMP expression measured by q-PCR. LCN2 secretion from (**c**) hMCs or (**d**) mMCs quantified by ELISA. (**e**) CAMP protein secretion from hMCs quantified by ELISA. *: *p* < 0.05, ****: *p* < 0.0001.

We measured these AMP mRNA expressions and protein secretions after PBS control or S1P stimulation in hMCs and mMCs (Figure 2a–e). As confirmed by q-PCR, our results revealed that after stimulating hMCs with S1P for 4 hrs, both LCN2 and CAMP expressions were increased (Figure 2a,b). To evaluate the protein release, hMCs and mMCs were stimulated with S1P for 24 h. ELISA data confirmed that both hMCs and mMCs released a significant amount of LCN2 after S1P stimulation (Figure 2c,d). We also confirmed that LCN2 production detected by ELISA was still increasing at 48 hr after hMC S1P stimulation (Figure S4). Likewise, we also confirmed that S1P induces a significant amount of CAMP after 24 h stimulation in hMCs (Figure 2e).

Next, we proved that bacterial encounters, especially gram-positive, are able to induce S1P from keratinocytes and MCs.

**Figure 3:**
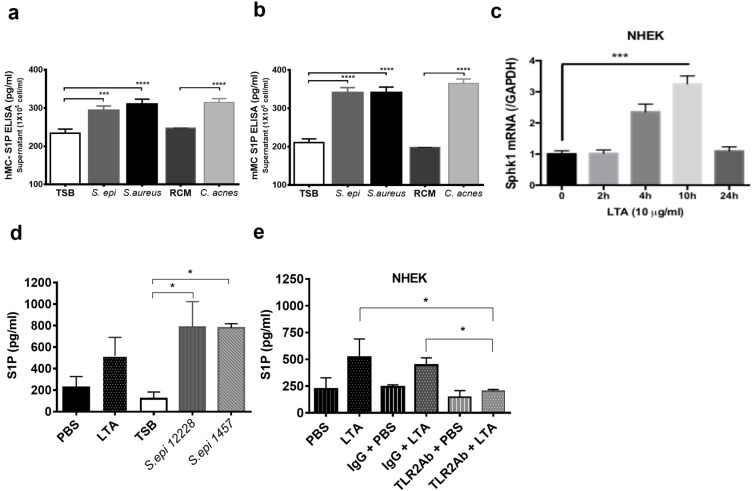
Both MCs and normal human epidermal keratinocytes (NHEKs) produce S1P against LTA or commensal bacterial supernatant stimulation (**a**) hMCs and (**b**) mMCs were stimulated with TSB (control for staphylococcus-bacterium), RCM (control for *C. acnes*), or 200 µL/mL bacterial supernatant from *S. epidermidis (S. epi)*, *S. aureus*, or *C. acnes* for 24 h, and S1P release from (**a**) hMCs or (**b**) mMCs were quantified by ELISA. (**c**) After 10 μg/mL LTA stimulation, NHEK *SPHK1* (*Sphingosine kinase 1)* expression was measured at different time points by q-PCR. (**d**) NHEKs were stimulated with PBS (control), 10 μg/mL LTA, 200 µL/mL *S. epidermidis* 12228, or 1457 for 24 h and S1P concentration was measured by ELISA. (**e**) NHEK TLR2 was blocked by 1 μg/mL TLR2 antibody (TLR2Ab) or IgG isotype control for 1 h. After TLR2 blocking, NHEKs were stimulated with 10 μg/mL LTA for 24 hr and S1P concentration was measured by ELISA. *: *p* < 0.05, ***: *p* < 0.001, ****: *p* < 0.0001.

S1P is known to be produced in large quantities by endothelial cells, which are frequently localized near mMCs [19]. However, MCs are also in the upper dermis where they could be stimulated by keratinocyte-derived S1P and by bacterial encounters that can induce an autocrine release of S1P. To confirm the significance of an S1P-MC autocrine release in response to bacteria, hMCs and mMCs were stimulated with different skin bacteria supernatants including *S. epidermidis* 12228, *S. aureus* SA113, and *C. acnes* 6919. S1P levels were subsequently measured with ELISA. The results demonstrated that all strains of bacteria increased S1P production in both hMCs (Figure 3a) and mMCs (Figure 3b). In addition, we also considered keratinocytes as a potential source of S1P. To confirm the production of S1P from keratinocytes, we first measured the expression of *Sphingosine kinase 1 (SPHK1)*, an essential kinase in the S1P biosynthetic pathway. After NHEKs were stimulated with LTA, *SPHK1* expression was measured at different time points. The results confirmed that *SPHK1* expression in NHEKs was increased at 4 and 10 h after LTA stimulation (Figure 3c). Next, S1P production was measured by ELISA. NHEKs were stimulated with LTA and whole supernatants from two different strains of commensal bacteria, *S. epidermidis* 12228, and *S. epidermidis* 1457. The results demonstrated that LTA and both of the commensal bacteria strains also increased S1P secretion from NHEKs (Figure 3d). To further investigate which receptor on NHEKs stimulates S1P release, NHEKs were blocked with an anti-TLR2 antibody before stimulation with LTA. ELISA results demonstrated that after blocking NHEKs with the anti-TLR2 antibody, the S1P release was significantly reduced (Figure 3e). These results suggest that bacteria on the skins surface not only induce an autocrine release of S1P from MCs, but also induce S1P secretion from NHEKs through TLR2, and that S1P will stimulate AMP release, such as LCN2, from MCs.

### 2.4. S1P Increase Antimicrobial Activity of hMCs and mMCs

**Figure 4:**
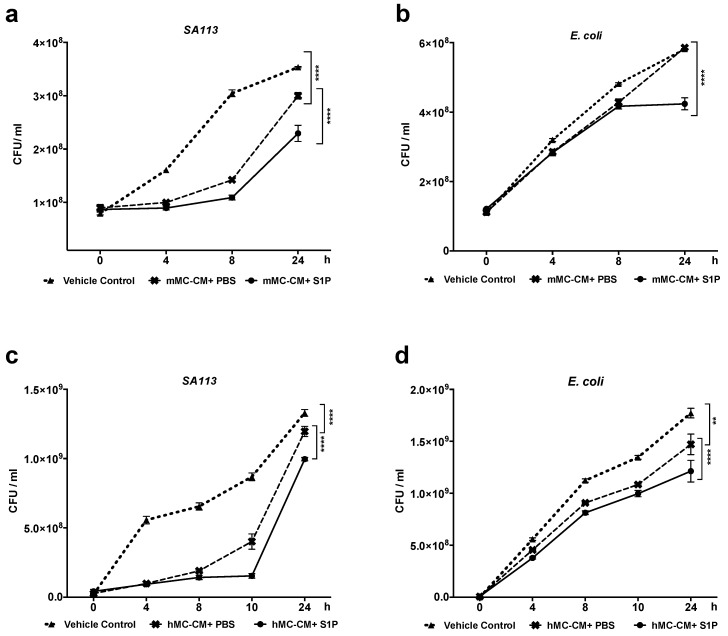
S1P activates and enhances MC antimicrobial activity. hMCs or mMCs were conditioned with either PBS or 200nM S1P in an antibiotic-free culture medium for 48 hr and conditioned mediums (hMC-CM+PBS or +S1P, mMC-CM+PBS or +S1P) were collected for antimicrobial liquid assays. (**a**–**d**) Antimicrobial liquid assay with (**a**,**b**) mMC-CMs or (**c**,**d**) hMC-CMs. (**a**,**c**) *S. aureus* SA113 or (**b**,**d**) *E. coli* were incubated in vehicle control (antibiotic-free cell culture medium), mMC or hMC-CM+PBS, or, mMC or hMC-CM+S1P. (**a**,**c**) *S. aureus* SA113 or (**b**,**d**) *E. coli* growths were demonstrated by CFU/mL. **: *p* < 0.01, ****: *p* < 0.0001.

To evaluate the effect of S1P on MC antimicrobial activity, both bone-marrow-derived mMCs and cord-blood-derived hMCs were stimulated with either PBS or S1P. Supernatants were collected as conditioned mediums (hMC- or mMC-CM+PBS or +S1P) 48 h after the stimulation. These conditioned mediums were used to perform antimicrobial liquid assays. mMC-CM+S1P showed significantly less growth of *S. aureus* SA113 when compared to the mMC-CM+PBS (Figure 4a). Additionally, the growth of *E. coli* was also significantly inhibited by mMC-CM+S1P (Figure 4b). Next, we confirmed that hMC antimicrobial activity against both *S. aureus* and *E. coli* were also enhanced by S1P (Figure 4 c,d).

hMC-CM+S1P has a higher capacity to inhibit *S. aureus* SA113 growth compared to hMC-CM+PBS (Figure 4c). Additionally, hMC-CM+PBS suppressed *E. coli* growth compared to vehicle control, while the hMC-CM+S1P suppressed even more *E. coli* growth compared to hMC-CM+PBS (Figure 4d). We also confirmed that without MCs, the S1P alone did not affect either *S. aureus* SA113 or *E. coli* growth (Figure S5). 

**Figure 5:**
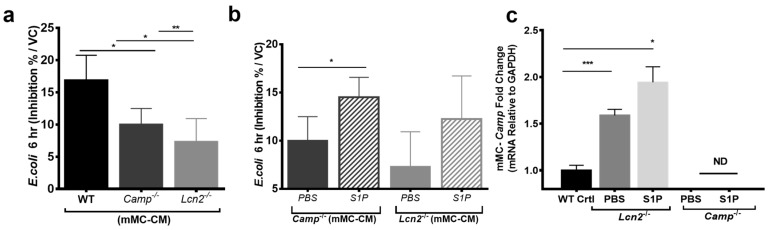
LCN2 is important for MCs capacity to defend against *E. coli*. mMCs derived from WT, *Camp*^-/-^, and *Lcn2^-/-^* mice were conditioned with PBS or 200nM S1P for 48 hr, and mMC-CMs were collected for antimicrobial liquid assays against *E. coli*. (**a**) Baseline antimicrobial activity of WT, *Camp*^-/-^ or *Lcn2^-/-^* derived mMC-CM against *E. coli* and (**b**) anti-*E. coli* inhibition activity of *Camp*^-/-^ or *Lcn2*^-/-^ derived mMC+PBS or +S1P. Bacteria growth was measured by OD600. Data was shown by *E.* *coli* inhibition % calculated by the formula shown in materials and methods. (**c**) *Camp* expression measured by RT-qPCR among mMCs derived from WT, *Lcn2*^-/-^ and *Camp^-/-^* mice with PBS or 200nM S1P stimulation. *: *p* < 0.05, **: *p* < 0.01, ***: *p* < 0.001.

Overall, our data indicate that S1P enhances both mMCs and hMCs antimicrobial capacity against *S. aureus* and *E. coli*. While these data showed that S1P increases MCs capacity to inhibit bacterial growth, our data did not address the relative importance of LCN2 as an AMP in MCs. Therefore, we compared the abilities to inhibit *E. coli* growth of mMC-CMs derived from the bone marrow of WT, *Lcn2*^-/-^ and *Camp*^-/-^ mice (Figure 5). The results demonstrated that the deficiency of *Lcn2* significantly diminished mMCs capacity to inhibit *E. coli* growth compared to the deficiency of *Camp* (Figure 5a). Next, we further conditioned mMCs derived from *Camp*^-/-^ and *Lcn2*^-/-^ mice with PBS or S1P and collected the conditioned media for additional antimicrobial liquid assays. Conditioning *Camp*^-/-^ and *Lcn2*^-/-^ mMC with S1P recovered mMC antimicrobial activities against *E. coli* (Figure 5b). We hypothesized that the residual recovery of *E. coli* growth inhibition in *Lcn2*^-/-^ mMCs after S1P treatment could have been due to Camp synthesis by S1P stimulation. Therefore, we evaluated *Camp* expression in *Lcn2*^-/-^ mMCs, and we found that *Lcn2*^-/-^ mMCs expressed more *Camp* than WT, even without S1P stimulation (Figure 5c). These experiments confirmed that Lcn2 is an important antimicrobial protein for protection against *E. coli* in mMCs. Moreover, this data further supports that S1P enhances antimicrobial activity and promotes Camp and Lcn2 production in mMCs.

## 3. Discussion

LCN2, also known as neutrophilic gelatinase associated lipocalin, is a member of the lipocalin superfamily and a mediator of many different inflammatory diseases. It is a 25kDa secreted glycoprotein that is encoded by a gene located at 9q34.11. LCN2 acts as an acute-phase-protein during bacterial infections [20] and exhibits a unique antimicrobial mechanism [21]. In fact, unlike traditional AMPs, LCN2 binds to bacterial siderophores to sequester iron from bacteria, ultimately inhibiting bacterial growth [12]. LCN2 activity is exerted by capturing bacterial siderophores produced by pathogens such as *E. coli*. In fact, *Lcn*2 deficient mice are more prone to infections and sepsis [12]. Studies have demonstrated that LCN2 synthesis is increased systemically during inflammation and infection in several different human diseases [22]. In addition, recent studies have shown that human LCN2 is expressed in the skin and is associated with wound healing and certain skin diseases, such as psoriasis [23].

In this study, we have shown that MCs produce and release LCN2. Given that MCs belong to the myeloid lineage [24] and that previous studies have demonstrated the expression of LCN2 in other myeloid cells, like macrophages, we predicted that MCs also express LCN2. To verify our hypothesis, we utilized deep-cadge RNA sequencing data from skin MCs and confirmed that human skin MCs expressed LCN2. In fact, skin MCs express an even higher amount of LCN2 compared to the in vitro (ex vivo-expanded) MCs in the FANTOM 5 transcriptomes study. Next, we confirmed that both hMCs and mMCs increase the synthesis of LCN2 in response to the TLR4 ligand, LPS. This is in agreement with previous studies that use other cell types to show increased LCN2 expression in response to *E. coli* [13]. TLR2 ligands, like LTA from gram-positive bacteria, are potent stimulators of CAMP production in MCs, and we demonstrated that LTA also induces Lcn2 expression and production, but only in mMC.

The LCN2 expression in MCs not only shows a wider involvement of hMCs in infections but also opens a new avenue of research for MCs role in psoriasis and wound healing, where LCN2 is highly expressed [23].

LCN2 binds at least two known surface receptors: LCN2 receptor (also known as 24p3R, NGALR, and SLC22A17) and megalin (also known as low-density lipoprotein receptor-related protein 2, LRP2), possibly interacting through these receptors with cellular iron metabolism and pro-inflammatory pathways via increased oxidative stress [22]. Oxidative stress is also linked to the S1P level in the cells [25]. S1P in the immune cell is essential for cell trafficking and its concentration is increased in many inflammatory conditions, such as asthma and autoimmunity [26]. Studies also suggest that S1P may act as an environmental signal that shapes the phenotype and functions of mast cells [27,28]. MCs express two out of the five receptors for S1P, namely S1PR1 and S1PR2 [29]. Recent reports have shown that in human keratinocytes, S1P acts upstream of NF-κB signaling, resulting in the direct binding of C/EBPa to the CAMP promoter, inducing its expression [30] and making S1P a promising mediator to also increase LCN2 AMPs in MCs. S1P is not only released by endothelial cells [31] but, as our data shows, NHEKs can also release a significant amount of S1P in response to LTA and bacterial products, either commensals or pathogens, (Figure 3c,d) with the prospective of conditioning nearby MCs to release AMPs in the dermis. The importance of TLR2-S1P-AMP interactions for MCs is emphasized by the fact that TLR2 is strictly downregulated in dermal MCs and the microbiome TLR2 signals are conveyed to MCs through keratinocytes and SCF production [16], as we demonstrated before, and now also by S1P.

In this present paper, we demonstrated that NHEK S1P production is dependent on TLR2 on NHEKs, since blocking TLR2 on NHEKs abolishes S1P production (Figure 3e), thereby showing that S1P is another way in which NHEKs convey the gram-positive microbiome signals to MCs in the dermis. Since S1P is a central signal in MC immune responses [32], we investigated whether S1P signaling could directly increase AMP production in MCs, especially LCN2, creating a possible alternative pathway to AMP activation. We can speculate that this is a way for commensal bacteria to maintain high levels of AMPs in MCs.

We also confirmed that S1P could be released from MCs themselves, as we have also described in a previous study [8].

Collectively, our data demonstrated that S1P increases the expression of both CAMP and LCN2 in MCs, significantly increases their release, as well as promotes antimicrobial activity against the gram-positive bacterium, *S. aureus,* and the gram-negative bacteria, *E. coli*. Although MCs express LCN2 without any stimulation, its expression is significantly increased after LPS and S1P stimulation. In addition to the classic TLRs-bacterial ligand, our study also shows the ability of S1P to stimulate the production of LCN2. Since it has been previously reported that the S1P-S1PR2 axis induces MC CAMP production [8], and that S1PR2 is also responsible for human and mouse MC degranulation and secretion of cytokines [33,34,35], we hypothesize that S1P mediated LCN2 secretion from MCs is caused by the same S1P-S1PR2 axis. In our future research, we will study in details the S1P-S1PR2 pathway. Though we could not completely elucidate the precise pathway of S1P mediated LCN2 synthesis in MCs, our finding indicates that the antimicrobial activity of MCs can be triggered even in the absence of TLR4 or TLR2 through LPS or LTA, respectively.

Because LCN2 contributes to the MCs antimicrobial capacity, we sought to evaluate the activity of LCN2 to inhibit *E. coli* growth by using MCs derived from *Lcn2^-/-^* mice. Our data indicates that LCN2 is important to MC activity against *E. coli.* These findings are in line with previous research showing that LCN2 plays a role in the acute phase response to *E. coli* in the intestine and kidney [13,36]. In line with this notion, we also demonstrated that mMC-CM derived from *Lcn2^-/-^* mice is less efficient for *E. coli* growth inhibition than mMC-CM derived from *Camp^-/-^* mice. Interestingly, the *Camp* gene expression is higher in *Lcn2^-/-^* mMCs than in WT mMC. However, despite the increased *Camp* expression in *Lcn2^-/-^* mMCs, this increase did not compensate for the loss of antimicrobial capacity in *Lcn2^-/-^* mMCs in *E. coli* growth inhibition. We interpreted this phenomenon as the AMP compensation mechanism. Previous research has shown that the genetic ablation of *Lcn2* can impair the mouse wound-healing process [37], while this report suggests that the lack of *Lcn2* could also change other functions of MCs rather than just their antimicrobial capacity. We will explore these functions in our future studies. While it is known that mMCs use AMPs to directly kill vaccinia viruses and GAS Streptococcus [7,8], this direct killing capacity through AMPs has not been confirmed before in hMCs. In this study, we have demonstrated that hMCs also have this anti-microbial capacity through AMPs.

In summary, we have identified a new antimicrobial protein LCN2 in human and mouse MCs. Moreover, we showed the role of S1P as a new messenger able to activate an antimicrobial response in MCs. S1P directly induces MCs to produce LCN2 in defense against bacteria. Our results propose a potential new pathway for antimicrobial activation in MCs that bypasses the traditional TLR pathway. The S1P signal released from various cell types in the skin, like keratinocytes, endothelial cells, and other immune cells, can justify the reasons why, despite the absence of skin MC TLR2 expression in vivo, MCs still produce CAMP and LCN2 against gram-positive bacteria invasion. Our future investigations will focus on the precise mechanism of this pathway, including the responsible receptor of S1P on MCs. Ultimately, our study confirmed LCN2 is directly linked to inhibiting *E. coli* growth in MCs, making it an important mechanism for the MCs antimicrobial activity in the innate immunity of the skin.

## 4. Materials and Methods

### 4.1. Mice

*Camp*^−/−^ on a C57BL/6J background, bred at our facility, and C57BL/6J wild-type control mice, were housed at the ACTRI at the University of California, San Diego (UCSD). *Lcn2*^−/−^ mice on a C57BL/6J background (a kind gift from Dr. Raffatellu at the University of California, San Diego) were used to generate mouse bone-marrow-derived mast cells. All animal experiments were approved by the UCSD Institutional Animal Care and Use Committee (S10288, 10/19/2016). 

### 4.2. Cells

Primary murine MCs (mMCs) were generated with our previously published protocol [8]. Briefly, we extracted bone marrow cells from the femurs of 5 to 8-week-old mice and cultured cells in RPMI 1640 medium (Invitrogen, Carlsbad, CA, USA), supplemented with 10% fetal bovine serum (Hyclone, Pittsburgh, PA, USA), 25 mM HEPES (pH 7.4), 4 mM l-glutamine, 0.1 mM nonessential amino acids, 1 mM sodium pyruvate, 50 μM 2-mercaptoethanol, 100 IU/mL penicillin, and 100 μg/mL streptomycin (Invitrogen, Carlsbad, CA, USA), with 1 ng/mL recombinant murine IL-3 (R&D Systems, Minneapolis, MN, USA) and recombinant 20 ng/mL murine stem cell factor (SCF) (R&D Systems, Minneapolis, MN, USA). After 4 weeks, MCs were consistently generated, as confirmed by the expression of CD117 (c-Kit) and the high affinity immunoglobulin E receptor (FcεRI). MCs maturation was confirmed by metachromatic staining with toluidine blue. The purity of mMCs were greater than 98%.

Human MCs (hMCs) were derived from human cord blood CD34+ cells (Astarte Biologics, Bothell, WA, USA), according to the protocol by Kirshenbaum and Metcalfe [38]. CD34+ cells were briefly cultured in serum-free culture media (Stemline II, Sigma, St. Louis, MO, USA) containing recombinant 100 ng/mL human SCF (R&D Systems, Minneapolis, MN, USA), 100 ng/mL recombinant human IL-6 (R&D Systems, Minneapolis, MN, USA), and 20 ng/mL recombinant human IL-3 first week only (R&D Systems, Minneapolis, MN, USA). After 10 weeks, hMCs were consistently generated, as confirmed by the expression of CD117 and FcεRI. Cell maturation was confirmed by metachromatic staining with toluidine blue. The purity of hMCs was greater than 98%.

Primary normal human epidermal keratinocytes (NHEKs, Life Technologies, Carlsbad, CA, USA) were grown in EpiLife medium (Life Technologies, Carlsbad, CA, USA) in accordance with the manufacturer’s instructions.

For other experiments, cells were treated with PBS control (Phosphate-buffered saline), TSB control (sterile 3 % tryptic soy broth, for staphylococcus-bacterium), RCM control (reinforced clostridial medium, for *C. acnes*), 10 μg/mL LTA (lipoteichoic acid, Sigma, St. Louis, MO, USA), 100 ng/mL LPS (lipopolysaccharide, Invivogen, San Diego, CA, USA), 200nM S1P (sphingosine-1-phosphate, Cayman Chemical, Ann Arbor, MI), or 200 µl/mL of 0.22 µm-filtered sterile flow (Millipore, Burlington, MA, USA) of different bacterial supernatants: *S. epidermidis* (ATCC12228 and ATCC1457), *S. aureus*-SA113 (ATCC35556), and *C. acnes* (ATCC 6919) according to previous reports [39,40,41]. Before LTA stimulation, NHEK TLR2 was blocked by 1 μg/mL TLR2 antibody or IgG isotype control for 1 h.

### 4.3. Evaluation of S1P Concentration in Cell Medium

Since our S1P was not prepared in Bovine serum albumin (BSA) containing PBS, we added 20 μg/mL of S1P to the cell cultures to obtain a working concentration of about 200nM of S1P. The actual S1P level of the treated medium with hMCs was evaluated by ELISA (MyBioSource, San Diego, CA, USA). The S1P ELISA data is reported in Figure S1a, and it shows that S1P is degraded at the 20 min interval.

We also confirmed that our S1P application did not affect human or mouse MC viability (Figure S1b,c). Viable cell number was counted using trypan blue.

### 4.4. Real-Time Quantitative RT-PCR

We used the RNeasy Mini Kit (QIAGEN, Hilden, Germany) to isolate total RNA (Ribonucleic acid) from cells. At least 1 µg of total RNA was used for cDNA synthesis by the iSCRIPT cDNA Synthesis Kit (Bio-Rad, Hercules, CA, USA) in accordance with the manufacturer’s instructions. A real-time quantitative polymerase chain reaction (RT-qPCR) was performed in an ABI 7300 Real-Time PCR system (Applied Biosystems, Foster, CA). All primers and probes used for the RT-qPCR were purchased from Applied Biosystems. RNA RT-PCR samples were prepared by using the TaqMan Master Mix reagents kit (Applied Biosystems). Gene expression analyses were calculated with the ΔΔ*C*T method to determine the quantification of gene expression. We normalized the target gene expression in the test samples to the levels of the endogenous reference, Glyceraldehyde 3-phosphate dehydrogenase (GAPDH), and reported them as the fold difference relative to GAPDH gene expression in untreated baseline controls [42]. All assays were performed with triplicated samples, and experiments were repeated more than two times.

### 4.5. ELISA

A human LL-37 ELISA kit (Hycult Biotech, Wayne, PA, USA and a human S1P ELISA kit (MyBioSource, San Diego, CA, USA) were used to determine LL-37 (the human cathelicidin peptide) and S1P levels in hMCs or NHEKs in accordance with the manufacturer’s instructions. Mouse and Human LCN2 ELISA kits (R&D Systems) were used for quantifying LCN2 protein secretion in both hMCs and mMCs.

### 4.6. Antimicrobial Liquid Assays

Antimicrobial liquid assays were performed as described previously [43]. Briefly, hMCs or mMCs were incubated in an antibiotic-free culture medium containing either PBS or 200 nM S1P. After 48 h of incubation, supernatants were collected and labeled: hMC- or mMC-conditioned medium+PBS, where hMC- or mMC-CM+PBS represents the supernatant from hMCs or mMCs incubated with PBS, and hMC- or mMC-CM+S1P represents the supernatant from hMCs or mMCs incubated with S1P, and vehicle control (VC) is an antibiotic-free culture medium. Log phase *S. aureus* (SA113, ATCC35556) and *E. coli* (a kind gift from Dr. Richard Gallo at the University of California, San Diego) were washed twice with PBS and resuspended to a concentration of 4 × 10 ^8^ CFU/mL (Colony-forming unit per milliliter) in Stemline II for hMC-assay or RPMI 1640 for mMC-assay. 25 μL of the prepared bacteria solution and 175 μL VC, MC-CM+PBS or MC-CM+S1P were mixed (total volume: 200 μL, total bacteria: 1 × 10 ^7^ CFU/well). The obtained bacteria-medium solutions were then incubated in a 96-well round-bottom tissue culture plate (Corning) for 10 or 24 h. We also prepared the antibiotic-free culture medium containing 200 nM S1P without any MCs, mixed it with the bacteria solution in the same ratio as mentioned above and incubated it for 24 h. All of the conditions were repeated three times for an OD600 measurement using a spectrophotometer (DTX880, Beckman Coulter, CA, USA). CFU of *S. aureus* and *E. coli* were calculated by the following formulas:

*S. aureus*: 1 OD600 = 3 × 10 ^8^ CFU/mL, *E. coli*: 1 OD600 = 3.5 × 10 ^8^ CFU/mL

Inhibition % was defined as the inhibition of bacterial proliferation due to mMC-CM+PBS or mMC-CM+S1P compared to VC and was calculated by the following formula in each time point (Target OD600: OD600 value of mixture incubated with VC or mMC-CM+PBS or mMC-CM+S1P derived from WT, *Camp^-/-^* or *Lcn2^-/-^* mice, VC OD600: OD600 value of mixture incubated with VC):

Inhibition % = {1 − (Target OD600/VC OD600)} × 100

### 4.7. Immunofluorescence Staining

mMCs were collected and concentrated on a microscopy slide by cytospin centrifuge. Cells were air-dried at room temperature and fixation/permeabilization solution kits (BD biosciences, San Jose, CA, USA) were used before staining. Purified anti-Lcn2 mouse antibody, clone: M0410B2 (Biolegend, San Diego, CA, USA), was used for primary staining overnight. Alexa Fluor 488 goat anti mouse IgG secondary antibodies (Thermo Fisher, Waltham, MA, USA) were applied for fluorescence conjugation. Incubation with secondary antibodies was only used as a negative control (Figure S2). Slides were mounted in ProLong Anti-Fade reagent with 4′-6-diamidino-2-phenylindole dihydrochloride (DAPI; Molecular Probes). Slide images were captured by an immunofluorescent microscope (Olympus, Center Valley, PA, USA)

### 4.8. Re-analysis of FANTOM 5 Data

Data were analyzed from FANTOM 5. We used MC transcriptome data from the FANTOM 5 data collection that was generated by an RNA-seq of hMCs freshly isolated from human skin and expanded in vitro [11] (http://fantom.gsc.riken.jp/5/suppl/Motakis_et_al_2013/) to investigate the innate immune phenotype of MCs in the skin. This transcriptome data was downloaded from the DNA Data Bank of Japan under accession numbers DRA000991 and DRA001026 (samples from human donors 1-4 (ex vivo MCs) and donors 5 and 8 (expanded in vitro MCs), respectively). This data includes the raw counts for all 184,827 DPI regions for 49 blood-related samples extracted from the FANTOM 5 data collection (the raw counts of the MC samples directly extracted from the skin and the raw counts of the MCs isolated from the skin and expanded in vitro for 5 weeks). The analysis of the data sets was performed by comparing the gene expression patterns of the ex vivo cells to the expanded (cultured cells), as a direct comparison of the TPM (Transcripts Per Kilobase Million) counts.

### 4.9. Statistical Analysis

All data are presented as the mean ± SD. At least three independent experiments were performed to assess the reproducibility of the different experiments. The *t*-test was used to determine significance between two groups, and one- or two-way ANOVA with Bonferroni’s post-test or Tukey’s multiple comparisons test were used to determine significance among multiple groups (GraphPad Prism, Version 5, GraphPad software, San Diego, CA, USA). For all statistical tests, *p* < 0.05 was considered statistically significant.

## Supplementary Materials

The following are available online at https://www.mdpi.com/1422-0067/20/10/2380/s1.
