# Supplementary figures and images for: Lipocalin 2: A New Antimicrobial in Mast Cells
Source: Int J Mol Sci. 2019 May 14;20(10):2380. doi: 10.3390/ijms20102380 (PMC6566617; doi:10.3390/ijms20102380)

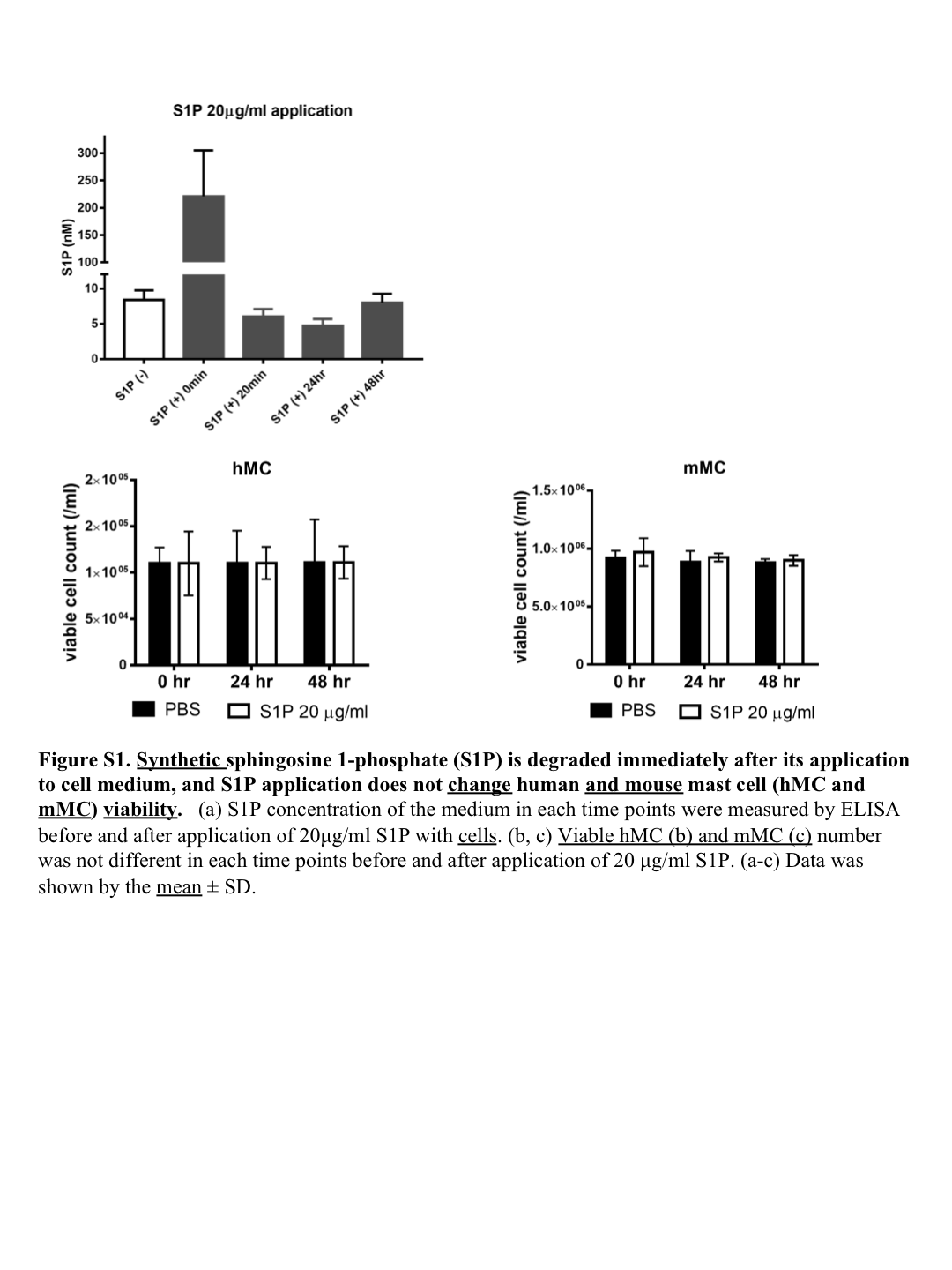

Supplement: Supplementary file 1 [file ijms-20-02380-s001.zip › Supplementary in Tiff 05_06_2019/Slide1.tiff]

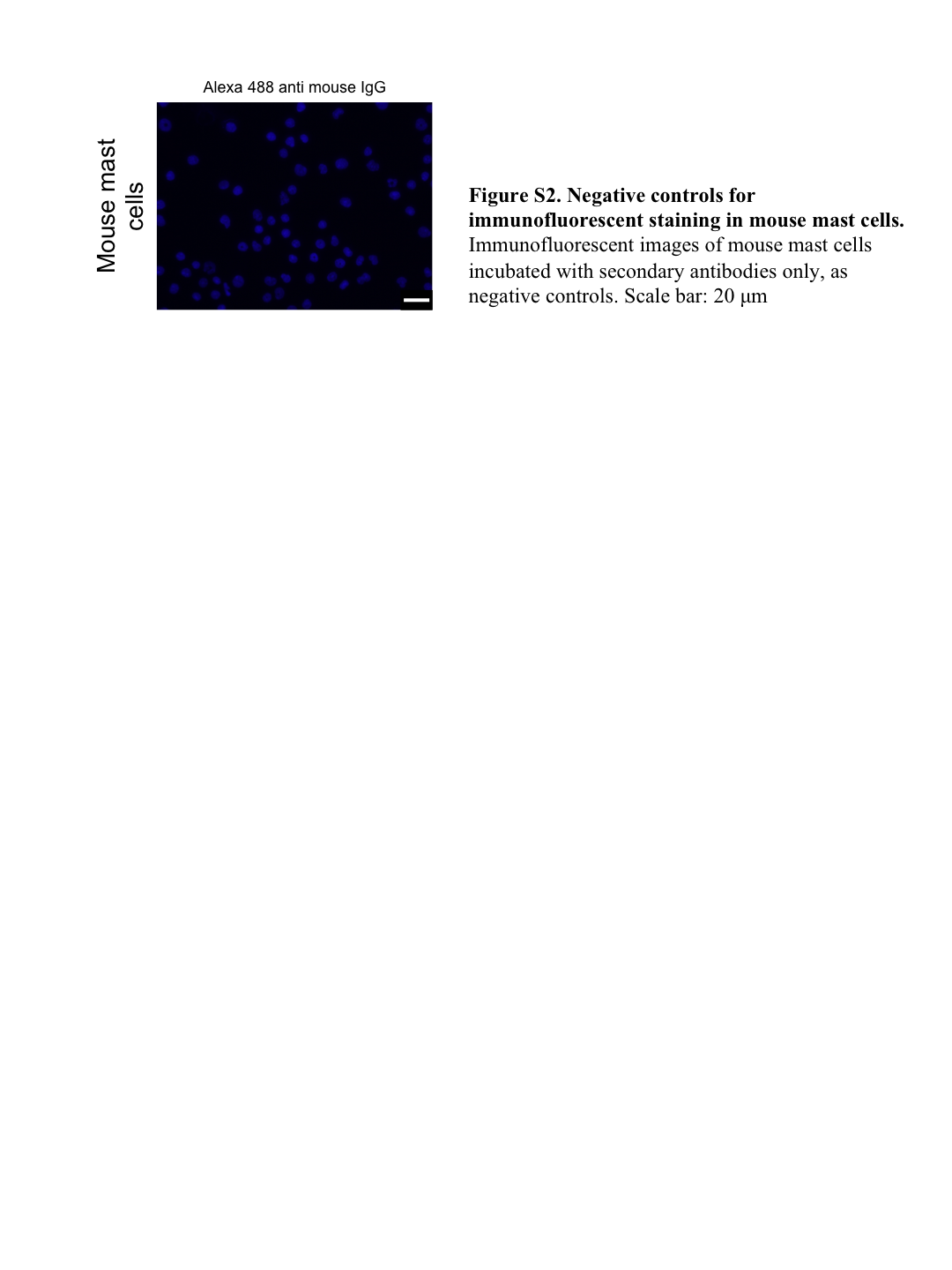

Supplement: Supplementary file 1 [file ijms-20-02380-s001.zip › Supplementary in Tiff 05_06_2019/Slide2.tiff]

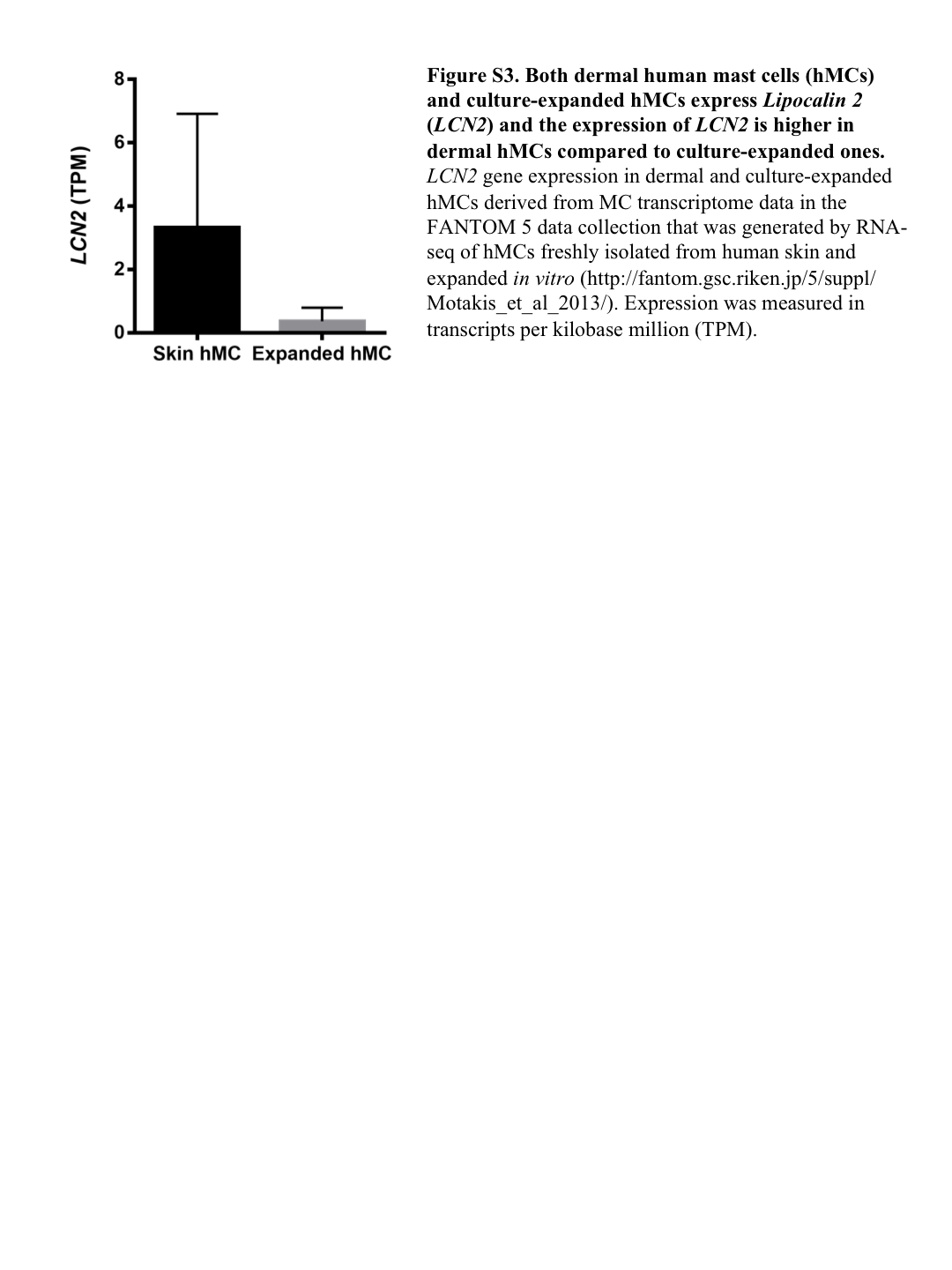

Supplement: Supplementary file 1 [file ijms-20-02380-s001.zip › Supplementary in Tiff 05_06_2019/Slide3.tiff]

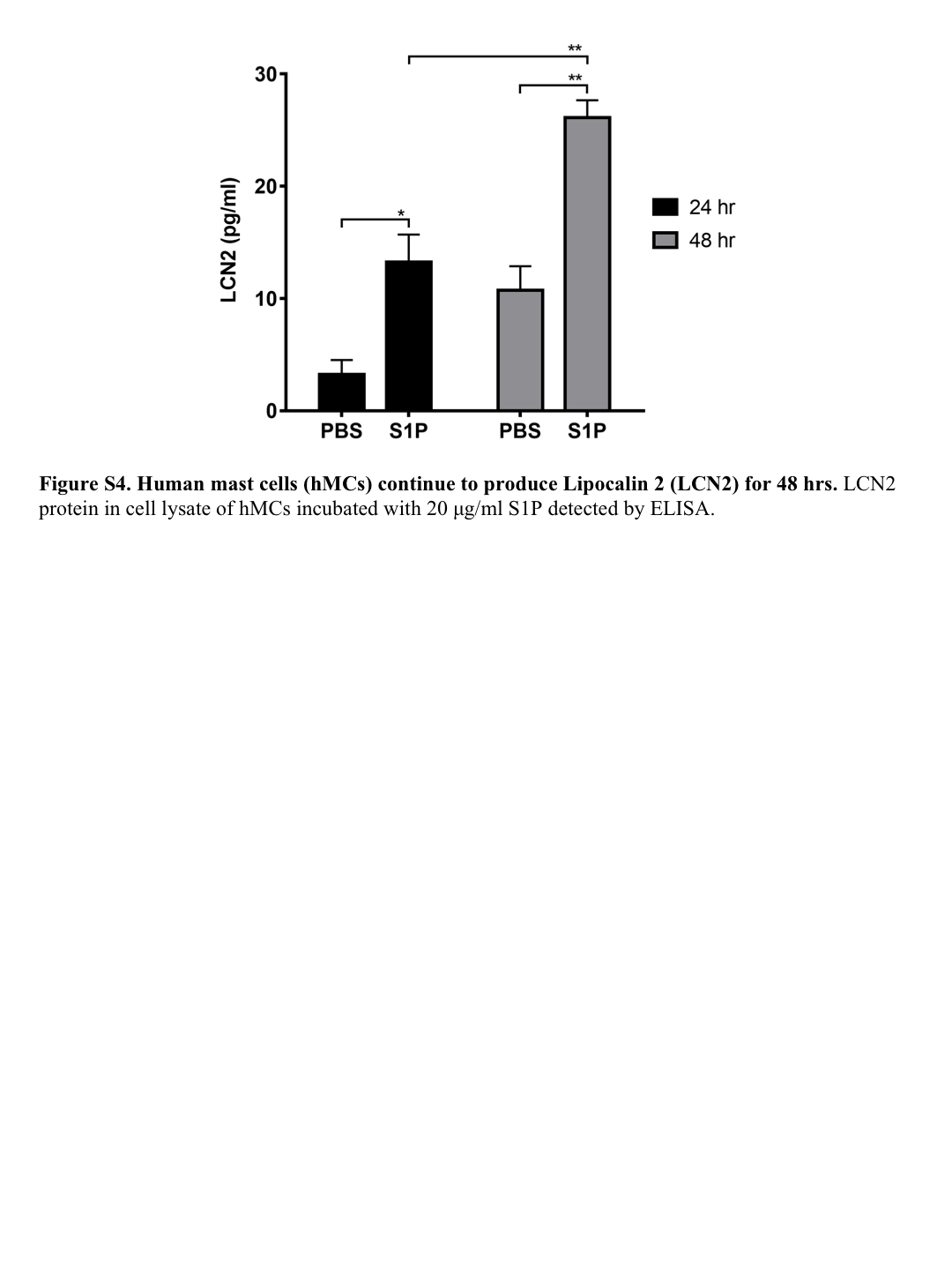

Supplement: Supplementary file 1 [file ijms-20-02380-s001.zip › Supplementary in Tiff 05_06_2019/Slide4.tiff]

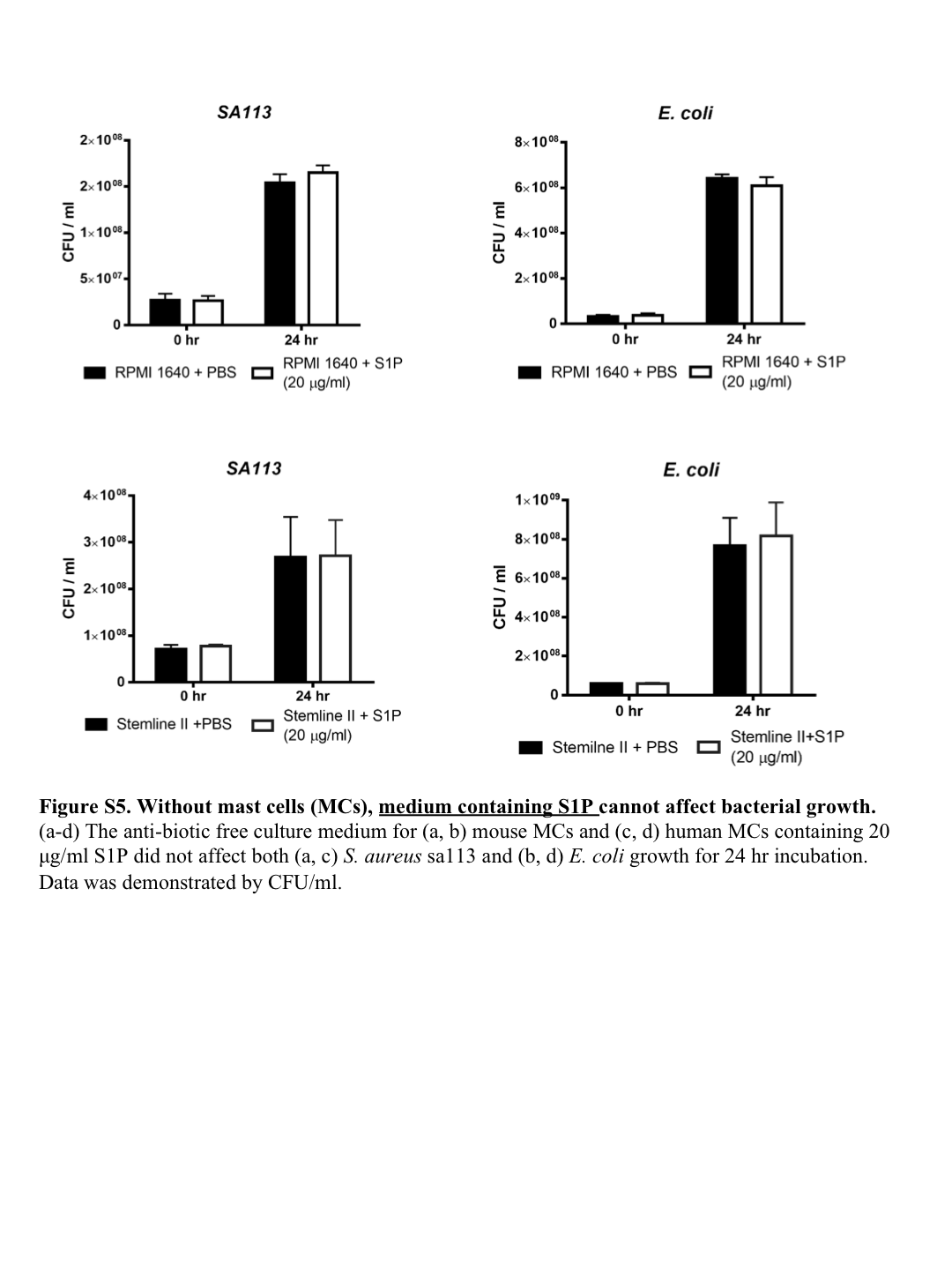

Supplement: Supplementary file 1 [file ijms-20-02380-s001.zip › Supplementary in Tiff 05_06_2019/Slide5.tiff]
